# Spatial analysis of completeness of death registration in Egypt

**DOI:** 10.1186/s42506-020-00040-3

**Published:** 2020-05-19

**Authors:** Nesma Lotfy

**Affiliations:** Department of Biostatistics, High Institute of Public Health, Alexandria University, Alexandria, Egypt

**Keywords:** Spatial analysis, Completeness, Death registration, Egypt

## Abstract

**Purpose:**

Civil registration and vital statistics (CRVS) systems should be the primary source of routine mortality data. However, there is lack of information about the completeness of death registration at the sub-national level of Egypt. The current study was conducted to estimate the completeness of death registration at the national and sub-national levels of Egypt, to investigate the spatial patterns of the completeness, and to examine the factors that influence it.

**Methods:**

Data from the Central Agency for Public Mobilization and Statistics (CAPMAS, 2018) and Egypt Demographic and Health Survey (EDHS 2008, 2014) were used to estimate the completeness of death registration using an empirical method (random-effects models); hot spot analysis was conducted using Moran’s *I* and Getis-Ord Gi*; and the geographically weighted regression (GWR) model has been also carried out.

**Results:**

The study estimates show that Egypt has 96% completeness of death registration, and all governorates have completeness of more than 90% except for Beni-Suef, Menia, Aswan, Suhag, Luxor, ELWadi ELGidid, and South Sinai. According to sex, the death registration of females is slightly better than that of males (96.8% compared to 95.4%). Concerning residence, urban area has almost complete death registration compared to rural area (99.5% and 85.4%, respectively). Hot spot analysis shows that all hot spots are centered on the north of Egypt, while all cold spots are focused on the south. However, according to the geographically weighted regression (GWR) model, poverty, illiteracy, and health office density are considered major factors for the completeness of death registration.

**Conclusion:**

Although the completeness in Egypt is almost 100%, this analysis suggests that it may not be, and that it could be somewhat lower in some rural areas. However, there is uncertainty in the sub-national estimates because deaths are only reported by place of occurrence and not place of usual residence. Thus, efforts should focus on improving the quality of data of the vital registration system in some rural areas and in lower Egyptian governorates.

## Introduction

Civil registration and vital statistics (CRVS) systems are the primary sources of accurate, timely, and regular data on deaths and other vital events for national and sub-national levels of government [1]. The data provided by CRVS systems is used for evaluating both national and sub-national level development plans as well as for monitoring the Sustainable Development Goals (SDGs). Well-operating CRVS systems are the best source of data for achieving 12 out of the 17 SDGs, and 67 out of the 232 SDG indicators [2].

Africa is a special case with respect to civil registration [3]. In many African countries, civil registration is still incomplete, despite decades of being in existence, and is therefore not being used as the major source of legal identity records and documents as well as for compiling vital statistics [4]. Only a few African countries have sustained a complete or near-complete vital registration for a long time. This is particularly the case for some North African countries including Egypt [5].

The vital statistics system in Egypt is a centralized system, where the Central Agency for Public Mobilization and Statistics (CAPMAS), under the Ministry of Planning, collects, compiles, and disseminates the vital statistics of the country. Health offices and units (under the Ministry of Health) register births and deaths at the local level and send these registration forms to the Civil Registration Office to be reviewed. Afterward, CAPMAS receives the registered vital events from the Civil Registration Office (under the Ministry of Interior) and then compiles and disseminates the statistics. CAPMAS also conducts population censuses and sample surveys to obtain vital rates [5, 6].

**Fig. 1:**
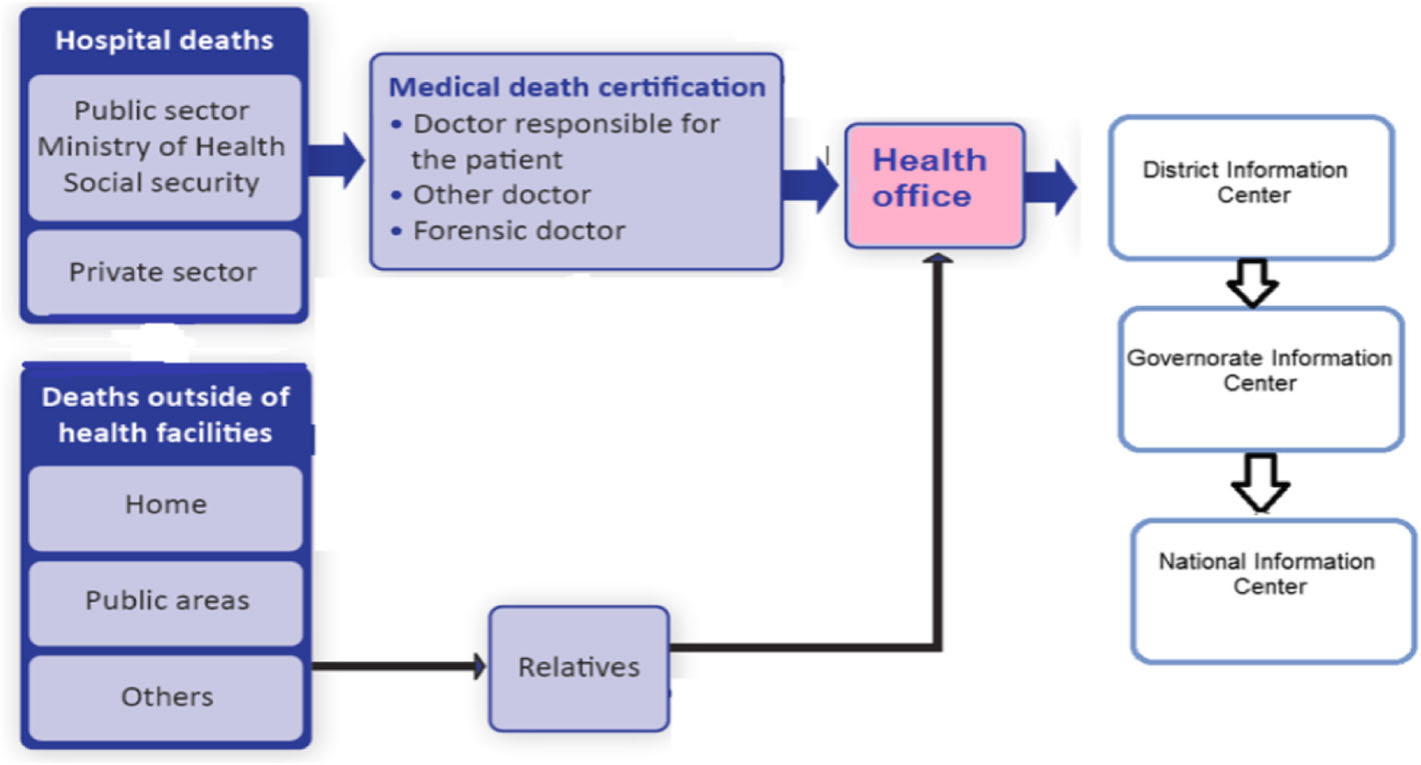
Death registration process in Egypt

Egypt has 27 governorates with 4571 local health units registering births and deaths and 284 health districts that reviewed the forms of births and deaths. The main feature of CRVS policies in Egypt is that the notification of death should take place within 24 h in the place of death in health offices. Figure 1 shows the data flow of death registration at health offices for either hospital or home deaths [7].

Estimation of the completeness of death registration can be calculated using three methods: indirect methods (death distribution methods (DDMs)), direct methods (capture-recapture methods), and comparing registered deaths to total deaths estimated using a range of data sources and methods, but each of these techniques suffer from several limitations [8].

Few studies have calculated the completeness of death registration in Egypt. For instance, a study by Silva [9] introduced three methods of death distribution methods (DDMs) (namely the generalized growth balance (GGB) method, the synthetic extinct generations (SEG) method, and the adjusted-SEG method) to estimate the sex differential in death registration completeness in Egypt. He found that the completeness of male death registration was 95%, 93%, and 80%, respectively, while completeness of female death registration was 88%, 92%, and 95%, respectively. On the other hand, using the estimated deaths of the Global Burden of Disease 2017 (GBD 2017) [10], the completeness of death registration in Egypt for both sexes was 109.5%, 95.1% for male and 134.5% for females. Another study [11] also measured the completeness of death registration through dual record systems. The analysis showed that the urban governorates (Cairo, Alexandria, Port Said, and Suez) had the highest percentage of completeness (98%), while the frontier governorates (Red Sea, ELWadi ELGidid, Matrouh, North Sinai, South Sinai) had the lowest percentage of completeness (56.2%).

Decades of lack of appropriate functioning of CRVS systems in many nations have resulted in that half of worldwide deaths are not registered [12]. Some of the reasons for incomplete vital registration data is that registration services are inaccessible to people living in rural areas and the quality of the services remains poor; therefore, the coverage and completeness of civil registration are very low in most of the rural areas [13].

Coverage and completeness of civil registration play an essential role for getting a reliable vital statistic; also, information on the registration completeness by geographic areas and demographic groups can be used to target interventions to strengthen CRVS and improve coverage. So, regular calculation of the completeness of death registration is needed at the national and sub-national levels. The aim of this study is to estimate the completeness of death registration at the national and sub-national level of Egypt, to investigate the spatial patterns of the completeness, and to examine the factors that influence it.

## Methods

### Study area

The study area is Egypt, which is located in North Africa. The GPS coordinates of Egypt are 26° 49′ 13.991″ N 30° 48′ 8.993″ E. The whole area of Egypt is about 1,000,000 km^2^. However, much of Egypt is desert, only 6.8% of its area is inhabited [14]. Administratively, Egypt is divided into 27 governorates, four of them are urban governorates (Cairo, Alexandria, Port Said, and Suez). Nine of these governorates are located in the Nile Delta (Lower Egypt) (Demietta, Dakahlia, Sharkia, Kalyoubia, Kafr EL Sheikh, Gharbia, Menoufia, Behera, Ismailia), nine are located in the Nile Valley (upper Egypt) (Giza, Beni-Suef, Fayoum, Menia, Asyout, Suhag, Qena, Aswan, Luxor), and the remaining five (Red Sea, ELWadi ELGidid, Matrouh, North Sinai, South Sinai) (frontier governorates) are located on the eastern and western boundaries of Egypt [15].

### Data management and population data source

The data sources were census [16], Annual Bulletin of Births and Deaths statistics [17], Income, Expenditure and Consumption survey [18], and Egypt in Figures [14] from Central Agency for Public Mobilization and Statistics (CAPMAS, 2018) and Egypt Demographic and Health Survey Datasets (EDHS 2008,2014) [19, 20]. The website from the Ministry of Planning, Monitoring and Administrative Reform (MPMAR) was used to get the number of heath offices for each governorate [21]. The calculation of the completeness was done by Excel. Spatial analysis was analyzed using the Esri ArcGIS 10.1 software.

### Estimating the completeness of death registration

Random-effects models were used to calculate the logit of death registration completeness at the national and sub-national level of Egypt which overcome the limitations of the other traditional methods. The models were developed from 2451 country-years in 110 countries (1970 to 2015) using the Global Burden of Disease 2015 database [8].

The models are as follow:

- For both sexes

$$ \mathrm{Logit}(X)=-0.0177\ast \mathrm{RegCDR}\mathrm{sq}+0.6375\ast \mathrm{RegCDR}+-13.89144\ast \left(\%65+\right)+-1.1136\ast \ln \left(5\mathrm{q}0\right)+2.2063\ast \mathrm{Compl}\ 5\mathrm{q}0+-0.0174\ast \mathrm{Year}+29.3677+\upgamma $$

- For male

$$ \mathrm{Logit}(X)=-0.0174\ast \mathrm{RegCDR}\mathrm{sq}+0.5957\ast \mathrm{RegCDR}+-12.9528\ast \left(\%65+\right)+-1.1266\ast \ln \left(5\mathrm{q}0\right)+2.0030\ast \mathrm{Compl}\ 5\mathrm{q}0+-0.0188\ast \mathrm{Year}+32.3442+\gamma $$

- For female

$$ \mathrm{Logit}(X)=-0.0198\ast \mathrm{RegCDR}\mathrm{sq}+0.6959\ast \mathrm{RegCDR}+-17.4154\ast \left(\%65+\right)+-1.1720\ast \ln \left(5\mathrm{q}0\right)+1.9387\ast \mathrm{Compl}\ 5\mathrm{q}0+-0.0144\ast \mathrm{Year}+23.5542+\upgamma $$

where

RegCDR is the registered crude death rate

RegCDRsq is the square of RegCDR

%65+ is the fraction of the population aged 65 years and over

ln(5q0) is the natural log of the estimated under-five mortality rate

Compl 5q0 is the completeness of the registered 5q0. This is calculated as the 5q0 from registration data divided by the estimated actual level of 5q0.

Year is a calendar year (2017)

γ is the random effect of Egypt (both sexes = 0.0907, male = 0.1982, female = 0.1291)

*X* is the completeness of registration at all ages

$$ \mathrm{Logit}(X)=\ln \left(\frac{X}{1-X}\right) $$

Estimation of under-five mortality can be done with different methods which require information obtained exclusively from censuses or surveys. One of them is known as the Brass method [22] which needs three detailed information: the number of children ever born, the number of children dead, and the total female population in the reproductive age (15 to 49, usually) [23]. Because of the lack of this information at census 2018, DHS rates R package [24] was used to estimate the under-five mortality rate (U5MR) for each governorate based on the DHS datasets using a reference period of 10 years preceding the survey of Egypt Demography and Health Survey 2008 and 2014 (EDHS 2008, 2014). The estimated U5MR was calculated by averaging the EDHS 2008 and 2014. After that, the estimated U5MR was scaled into the Inter-agency Group for Child Mortality Estimation (IGME) under-five mortality rates in Egypt for 2017. South Sinai had no values for the estimated U5MR, so the value of the adjacent governorate that is North Sinai was used.

### Spatial analysis

#### Hot spot analysis

Moran’s *I* was used to define the spatial autocorrelation and it ranges from − 1 to + 1. If Moran’s *I* is near to + 1, strong spatial autocorrelation occurs, which means that values cluster together. On the other hand, weak spatial autocorrelation occurs when Moran’s *I* is near to − 1, which means dissimilar values occur next to each other. Moran’s *I* can be converted into a *Z* score to test the statistical significance of spatial autocorrelation [25].

Getis-Ord Gi* spatial statistics tool was used to detect clusters of both hot spots (high values) and cold spots (low values). A hot spot happens when a high value is surrounded by other features with high values as well, while the cold spot happens when a low value is surrounded by other low values [26].

#### Modeling spatial relationships

Ordinary least square (OLS) regression and geographically weighted regression (GWR) [27] using the fixed Gaussian model were performed to explore the spatial relation between the completeness of death registration and explanatory variables (poverty, illiteracy, health offices density (number of health offices per 100,000 population)). The corrected Akaike information criterion (AICc) [28] was used in comparison between the two models. If the difference in the AICc values between OLS model and GWR model is more than 3, the GWR model can be considered more appropriate than the OLS model, even though it is more complex [29]. The GWR model has been found superior to the OLS model, so the results of GWR will be only represented (Appendix 1).

## Results

### Completeness of death registration

The results show that Egypt has 96% completeness of death registration (Table 1). With the exception of Beni-Suef, Menia, Aswan, Suhag, Luxor, ELWadi ELGidid, and South Sinai; all other governorates have more than 90% completeness of death registration.

**Table 1:** Completeness of death registration of 27 governorates by sex, residence, and overall of Egypt, 2017

Governorate	Sex		Residence		Total
	Male	Female	Urban	Rural	
Egypt	95.4	96.8	99.5	85.4	96.0
Cairo	99.6	99.9	99.8		99.8
Alexandria	99.7	99.5	99.7	86.9	99.7
Port-Said	96.5	99.4	98.1		98.1
Suez	99.1	98.9	99.1		99.1
Demietta	99.1	99.7	100.0	94.0	99.4
Dakahlia	97.1	98.6	97.4	98.1	97.9
Sharkia	91.1	93.8	95.9	90.4	91.8
Kalyoubia	89.9	98.7	99.3	87.3	94.3
Kafr EL sheikh	91.2	97.1	100.0	82.6	94.0
Gharbia	97.5	97.8	100.0	91.2	97.6
Menoufia	98.4	99.2	100.0	92.5	98.9
Behera	91.9	95.7	100.0	81.6	93.4
Ismailia	91.4	97.1	99.5	62.5	93.9
Giza	96.3	97.8	98.8	91.4	97.0
Beni-Suef	83.4	90.7	94.5	80.8	86.3
Fayoum	90.9	95.2	100.0	82.7	92.9
Menia	86.6	86.1	99.8	73.6	85.1
Asyout	94.8	94.0	99.8	68.9	94.1
Suhag	82.1	87.4	96.8	75.5	83.4
Qena	89.5	93.6	99.8	82.7	91.1
Aswan	81.7	92.6	93.4	76.5	85.5
Luxor	82.0	92.6	97.3	64.0	87.1
Red Sea	96.2	88.1	95.8	3.7	92.9
ELWadi ELGidid	96.2	69.8	97.5	78.3	83.4
Matrouh	93.9	91.0	95.1	75.5	92.0
North Sinai	95.0	88.2	98.0	67.1	92.8
South Sinai	91.4	83.6	97.8	19.3	88.2

According to sex, the death registration of females is slightly better than that of males (96.8% compared to 95.4%, respectively). However, at the sub-national level, the completeness of females’ death registration is very low compared to males at the frontier governorates such as Red Sea, ELWadi ELGidid, North Sinai, and South Sinai. Meanwhile, males’ death registration is very low at Beni-Suef, Qena, Aswan, and Luxor compared to females.

Concerning residence, the urban areas have almost complete death registration compared to rural (99.5% and 85.4%, respectively). However, in rural areas, there is a great variation of completeness of vital registration at the sub-national level, only 6 governorates have more than or equal to 90% completeness (Demietta, Dakahlia, Sharkia, Gharbia, Menoufia, and Giza). In contrast, Ismailia, Asyout, Luxor, and North Sinai, have less than 70% completeness, while Red Sea and South Sinai have the lowest completeness (3.7% and 19.3%, respectively).

Table 2 shows the completeness of death registration according to region. As expected, the completeness of death registration is higher in urban areas than rural, and in Lower Egypt than Upper Egypt. On the other hand, in rural Lower and Upper Egypt, female’s death registration is higher than males.

**Table 2:** Completeness of death registration according to region by sex, Egypt, 2017

Area	Deaths		
	Male	Female	Total
Urban governorates	99.6	99.8	99.7
Lower Egypt	94.7	97.6	96.1
Urban	99.5	99.8	99.7
Rural	86.9	94.3	89.9
Upper Egypt	90.3	92.5	90.8
Urban	99.0	99.0	99.1
Rural	75.9	84.7	78.4
Frontier governorates	97.8	91.6	95.7

### Spatial analysis

#### Hot spot analysis

**Fig. 2:**
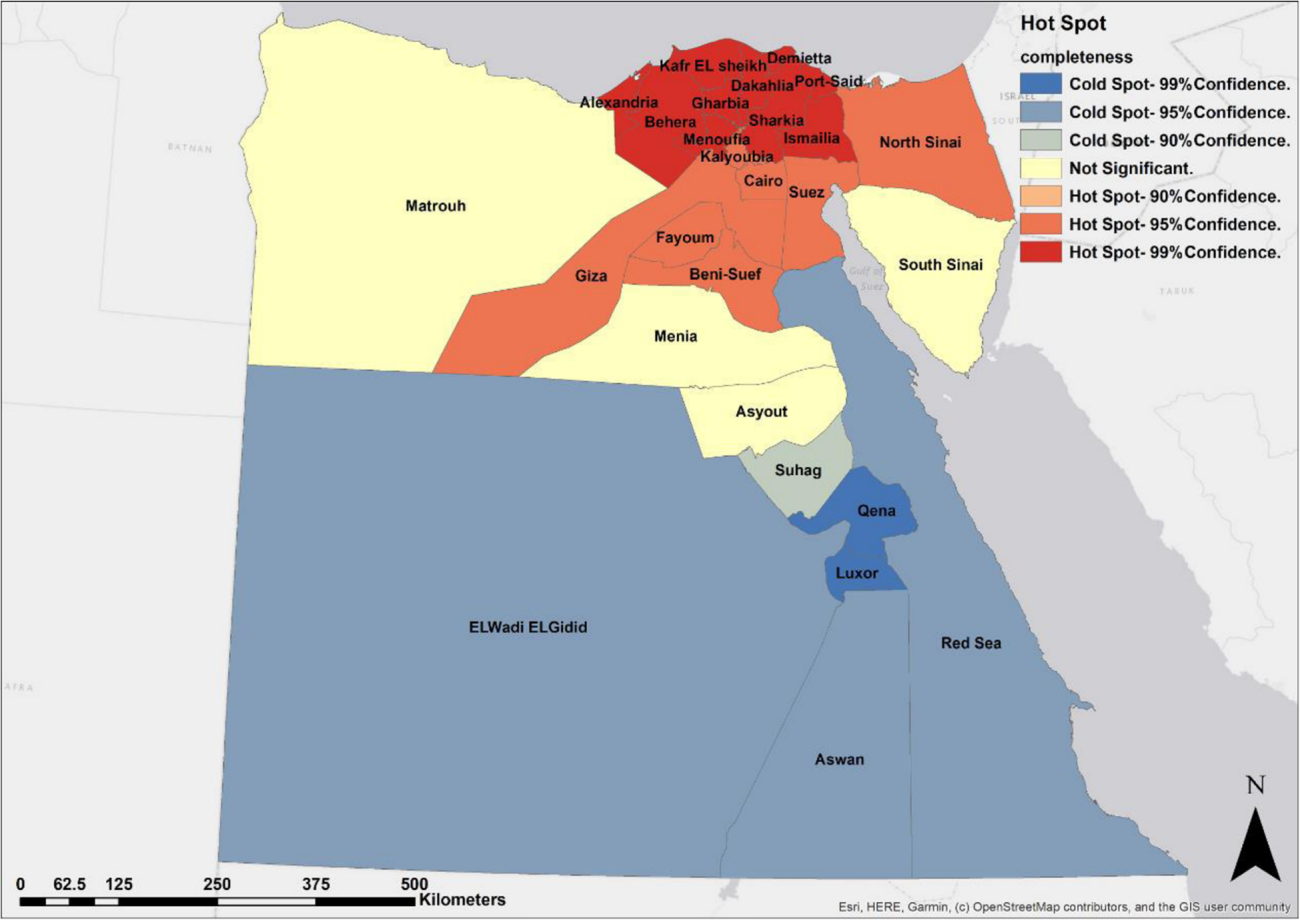
Hot spots of the completeness of death registration, Egypt, 2017

Results from Moran’s *I* reveal that the spatial distribution of the completeness of death registration at the sub-national level is spatially clustered regarding all Egypt, sex, and residence. Figure 2 indicates the hot spot analysis of the completeness of death registration in Egypt, 2017. It shows that the most significant hot spots are found at 10 governorates (Alexandria, Kafr EL Sheikh, Gharbia, Behera, Menoufia, Dakahlia, Demietta, Port-Said, Ismailia, and Sharkia), while the second significant hot spots at 7 governorates (Cairo, Kalyoubia, Giza, Fayoum, Beni-Suef, Suez, and North Sinai). On the other hand, the most significant cold spots are found at 2 governorates (Luxor and Qena), while the second significant cold spots at 3 governorates (Aswan, Red Sea, and ELWadi ELGidid). Appendix 2 shows the hot spots of completeness for males and females, respectively.

According to urban, spatial pattern of completeness is observed as hot spots in 19 governorates and cold spots in 2 governorates (Luxor and Aswan). In contrast, rural areas, 6 governorates classified as hot spots (Alexandria, Matrouh, Giza, Fayoum, Beni-Suef, and Menia) compared to 5 governorates classified as cold spots (Suhag, Qena, Luxor, Red sea, and Aswan) (Appendix 2).

### Modeling spatial relationships

#### Geographically weighted regression

##### Spatial autocorrelation

Determining whether the variables are spatially autocorrelated is needed before implementing the GWR model. The Moran’s *I* autocorrelation values indicated that the variables have positive spatial autocorrelations (Table 3).

**Table 3:** Moran’s *I* test for spatial autocorrelation of all variables

Variables	Moran’s *I*	*p* value
Completeness (%)	0.243	0.000*
Poverty (%)	0.386	0.000*
Illiteracy (%)	0.067	0.029*
Health offices density (per 100,000 person)	0.068	0.024*

*Significant at 0.05

##### GWR model

Table 4 presents the results of the estimate coefficients from the GWR model which are displayed as min, max, mean, and SD. The residuals of GWR model are randomly distributed (Moran’s *I* = − 0.053, *p* = 0.762).

**Table 4:** Summary of GWR model results

Local (GWR)					
Variables	**Min**	**Max**	**Mean**	**SD**	**Moran’s*I*for residual**
Intercept	104.34	107.27	106.2	0.693	− 0.053(0.762)
Poverty (%)	− 0.14	− 0.083	− 0.105	0.016	
Illiteracy (%)	− 0.303	− 0.149	− 0.261	0.044	
Health offices density (per 100,000 person)	− 0.721	− 0.314	− 0.436	0.093	

## Discussion

This paper provides an exploratory analysis for the completeness of death registration in Egypt. It indicated that Egypt has a near-complete death registration, almost 96% for both sexes, 95.4% for males, and 96.8% for females which is nearly equivalent with completeness estimated from GDB 2017 (109.5%, 95.1%, and 134.5%, respectively) [10]. Moreover, the synthetic extinct generations (SEG) method used by Silva [9] gave an estimate of completeness which is approximately close to the current method (SEG: male 93%, female 92%; empirical method: male 95.4%, female 96.8%). On the other hand, generalized growth balance (GGB) method and adjusted-SEG method reported different figures than ours (GGB: male 95%, female 88%; adjusted-SEG: male 80%, female 95%).

The study found that the rural area has the smallest completeness relative to the urban area, which may result from many reasons; deaths are registered according to the place of occurrence and not by residence [7], and health offices may be inaccessible (far away) [13].

By comparing the completeness of death registration in 1975 [11] with 2017, the completeness of death registration has been improved in almost all regions, but in Rural, Lower Egypt, the completeness has declined by 2.4% for male death registration, and it has slightly decreased by 0.4% for both sexes. For frontier governorates, the completeness has dramatically increased by more or less 40% for males, females, and both sexes.

The present work is the first study on spatial analysis of the completeness of death registration in Egypt. Conducting this research is highly needed to identify the hot spots and cold spots of the completeness of death registration to help decision makers in the vital registration systems. In this study, Moran’s *I* tool confirmed the existence of completeness clusters. Hot spot analysis using Getis-Ord Gi* was conducted in order to identify those clusters. It was found that all the hot spots are located at 17 governorates in the north of Egypt, while all the cold spots are located at 6 governorates in the south which are Luxor, Qena, Aswan, Red Sea, ELWadi ELGidid, and Suhage.

According to the GWR model, poverty, illiteracy, and health offices density are founded to be the most important factors associated with the completeness, and they can clearly explain the variations in the completeness of death registration. The model demonstrated that poverty and illiteracy showed a significant negative association with the completeness of death registration. Similarly, there is a significant negative association with the health offices density. Since the deaths are registered according to the place of occurrence and not by residence, we can conclude that most of registrations will occur at urban areas because of rural urban migration (searching for employment opportunities, education, and health care) [30]. So, increasing the number of health offices at rural areas will not support the completeness of death registration.

### Limitations of the study

The present study has several limitations: First, there is uncertainty around the estimated U5MR that can affect the model results. Second, the empirical method used is more accurate at the national level and less accurate at the sub-national level. Finally, deaths are registered according to the place of occurrence and not by the place of residence resulting in increase of the completeness in urban areas.

## Conclusions and recommendations

Although the completeness of death registration in Egypt is found to be very high (almost 100%), this analysis suggests that it may not be universally so and that it could well be somewhat lower in some rural areas. However, there is uncertainty in the sub-national estimates because deaths are only reported by place of occurrence and not place of usual residence. Thus, efforts should focus on improving the quality of data of the vital registration system in some rural areas and in lower Egyptian governorates.

## Figures and Tables

**Fig. 3:**
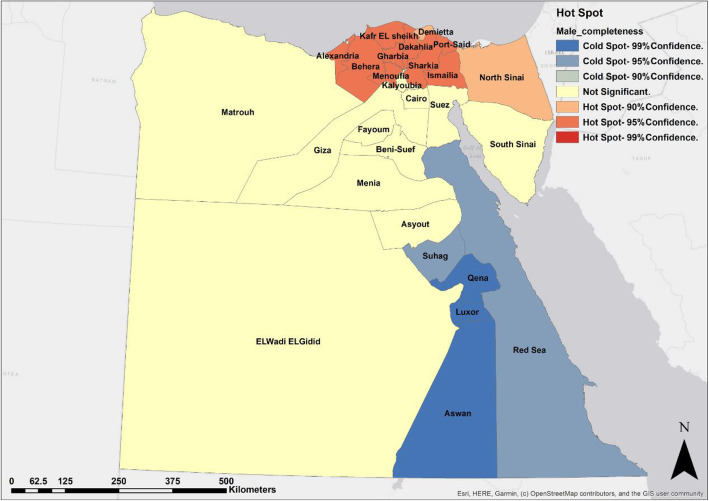
Hot spots of the completeness of male death registration, Egypt, 2017

**Fig. 4:**
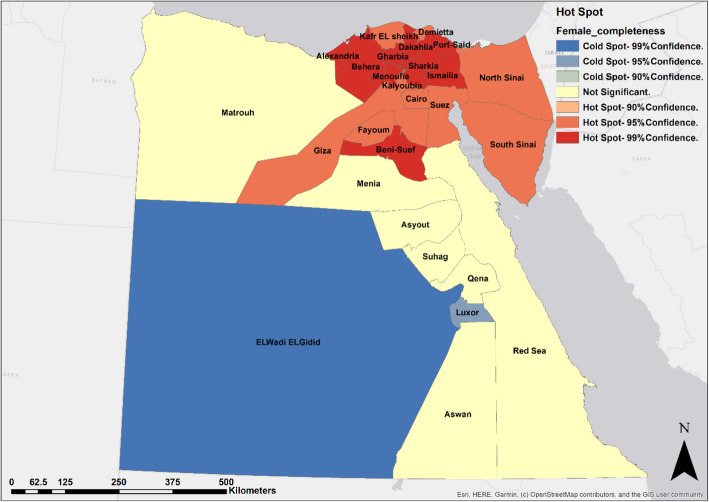
Hot spots of the completeness of female death registration, Egypt, 2017

**Fig. 5:**
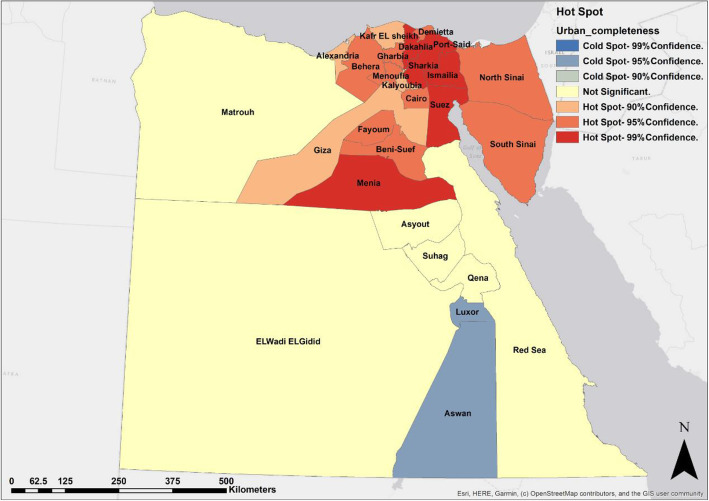
Hot spots of the completeness of urban death registration, Egypt, 2017

**Fig. 6:**
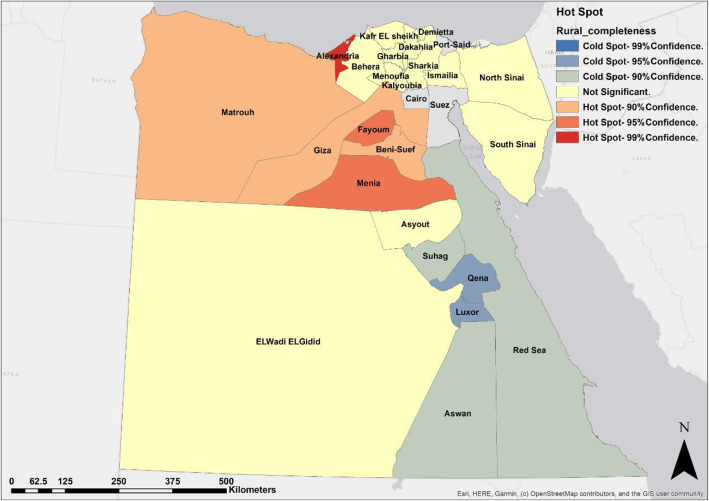
Hot spots of the completeness of rural death registration, Egypt, 2017

## Data Availability

All data are available on reasonable request.

## Appendix 1

By comparing the two models, the value of the AICc is reduced from 151.73 (OLS model) to 147.53 (GWR model). The difference is 4.2 implying that the GWR model fitness is better than OLS model. The adjusted *R*^2^ of the GWR model has increased about 10% (GWR = 0.665, OLS = 0.567), which demonstrated that the GWR has strongly explained the variance of the dependent variable compared to OLS model. Thus, the results show that the GWR model works well rather than OLS model (Table 5).

**Table 5:** Model fitness comparison

Fitness parameters	OLS	GWR
AICc	151.73	147.53
*R*^2^	0.617	0.74
Adjusted *R*^2^	0.567	0.665
